# Flavonoids in *Juglans regia* L. Leaves and Evaluation of *In Vitro* Antioxidant Activity via Intracellular and Chemical Methods

**DOI:** 10.1155/2014/303878

**Published:** 2014-07-15

**Authors:** Ming-Hui Zhao, Zi-Tao Jiang, Tao Liu, Rong Li

**Affiliations:** ^1^Tianjin Key Laboratory of Food Biotechnology, College of Biotechnology and Food Science, Tianjin University of Commerce, Tianjin 300134, China; ^2^College of Science, Tianjin University of Commerce, Tianjin 300134, China

## Abstract

Flavonoids are rich in *Juglans regia* L. leaves. They have potent antioxidant properties, which have been related to regulating immune function and enhancing anticancer activity. Herein, qualitative and quantitative determination of flavonoids from *J. regia* leaves was carried out using high performance liquid chromatography coupled with tandem mass spectrometry with electrospray ionization and negative ion detection (HPLC-ESI-MS/MS) by comparison of the retention times and mass spectral fragments with standard substances or related literatures. Seventeen compounds were identified and major components are quercetin-3-O-rhamnoside (453.11 *μ*g/g, dry weight), quercetin-3-O-arabinoside (73.91 *μ*g/g), quercetin-3-O-xyloside (70.04 *μ*g/g), kaempferol-O-pentoside derivative (49.04 *μ*g/g), quercetin-3-O-galactoside (48.61 *μ*g/g), and kaempferol-O-pentoside (48.46 *μ*g/g). The *in vitro* intracellular antioxidation indicated that flavonoids from *J. regia* leaves could reduce the reactive oxygen species (ROS) level in RAW264.7 cells and showed good radical scavenging activities. These results proved to be more related to the flavonoids that could be considered in the design of new formulations of dietary supplements or functional foods.

## 1. Introduction

*Juglans regia* L. leaves are good sources of flavonoids. Flavonoids have potent antioxidant properties, which have been related to regulating immune function and enhancing anticancer activity. The plant belonging to the genus of* Juglans* from* Juglandaceae* family is a deciduous tree and native to the region stretching from the Balkans eastward to the Himalayas and southwest China. Now it is widely distributed in Asia, southern and eastern Europe, and the United States. Its fruit is a valuable and nutritional nut, whose oil is rich in unsaturated fatty acids, tocopherols, and phytosterols [1].* J. regia* leaves are also used as a traditional medicine in China and Europe and have shown various health benefits for the treatment of skin inflammations, venous insufficiency, and ulcers. Moreover, the researches in pharmacology and therapeutics have shown that* J. regia* leaves have hypoglycaemic, antioxidative, antimicrobial, and antihypertensive effects [2, 3].

Recently, a large number of researches are mainly focused on the extraction/isolation and the antioxidant effect of flavonoids. Regarding* J. regia *leaves, there are a few reports on polyphenolic compounds of* J. regia* leaves from Portuguese cultivars. In the previous studies [1, 4, 5], four flavonoids and five phenolic acids were identified including quercetin-3-O-galactoside, auercetin-3-O-arabinoside, auercetin-3-O-xyloside, auercetin-3-O-rhamnoside, 3- and 5-O-caffeoylquinic acids, 3- and 4-*p*-coumaroylquinic acids, and* p*-coumaric acid. In addition, two flavonoids were partially identified such as quercetin-3-O-pentoside derivative and kaempferol-3-O-pentoside derivative. In addition, the extract of Portuguese cultivars can significantly inhibit the growth of Gram-positive bacteria, in particular* Bacillus cereus,* and shows some antioxidant activities [5, 6]. However, to the best of our knowledge, there is no report on flavonoids of* J. regia *grown in other countries and regions including China. Due to the influences of geographical environment, temperature, exposure time, rainfall, and other factors, the same plant grown in different countries will be very different in the chemical composition. Therefore, it is also necessary to study the flavonoids of* J. regia* leaves grown in China.

This study aimed to qualitatively and quantitatively determine the flavonoids of native* J. regia* leaves by HPLC-ESI-MS/MS and hoped to provide a reliable basis for use of this plant resource. Ten compounds are first found among all identified flavonoids from* J. regia*. Further, the antioxidant activities of flavonoids of native* J. regia* leaves were evaluated using intracellular and chemical methods.

## 2. Materials and Methods

### 2.1. Plant Material

The leaves of* J. regia*, which were identified by plant taxonomist Dr. Xuchun Wang, were collected from Tianjin, China, in August 2012. The leaves were dried at 60°C in a vacuum drying oven, ground to 60–80 mesh size, and stored at room temperature in a desiccator until use.

### 2.2. Chemicals

Epicatechin, quercetin-3-O-galactoside, and quercetin-3-O-rhamnoside standards were purchased from Shunbo Co. (Shanghai, China). 5-O-Caffeoylquinic acid (neochlorogenic acid), quercetin-3-O-arabinoside, and quercetin-3-O-glucuronide standards were obtained from Yifang S & T Co. (Tianjin, China). All standards were at least 98% purity.

2′,7′-Dichlorofluorescin diacetate (DCFH-DA) was purchased from Beyotime Institute of Biotechnology (Shanghai, China). 2,2-Diphenyl-1-picrylhydrazyl (DPPH), dimethyl sulfoxide (DMSO), and 3-(4,5-dimethylthiazol-2)-2,5-diphenylterazolium bromide (MTT) were purchased from Sigma-Aldrich (Shanghai, China). Methanol was purchased from Merck (Darmstadt, Germany). Formic acid was purchased from Tedia Co. Inc. (Fairfield, OH, USA). All the chemicals used were of analytical or HPLC grade. Deionized water was purified by a Milli-Q system (Millipore, Bedford, MA, USA) and used throughout.

RAW264.7 cells (Monocytic leukemia cell mouse macrophage) were obtained from American Type Culture Collection (Manassas, VA, USA). AB-8 macroporous adsorption resin was obtained from Nankai Hecheng S & T Co. (Tianjin, China).

### 2.3. Preparation of Extract from Leaves of* J. regia*

Ethanol solution (60%, v/v) was used for the extraction. Firstly, 5 g of leave samples was placed in a microwave vitreous flask and extracted with 175 mL of 60% ethanol. The flavonoids and other polar compounds were extracted by microwave treatment for 7 min with a microwave power of 400 W at 85°C. Then, the extract was filtered and the fat-soluble components in the filtrate were removed using isopyknic petroleum ether. Finally, the ethanol extract was concentrated in a rotary evaporator to remove ethanol and then freeze-dried. Thereby, the lyophilized extract from* J. regia* leaves was obtained.

### 2.4. Purification of Extract from Leaves of* J. regia*

The obtained extract contains not only flavonoids but also other polar compounds as well as other impurities. In addition, the content of some flavonoids is very low. In order to identify such low levels of the compounds to improve the accuracy of the analysis, it is necessary to purify the extract by use of AB-8 macroporous adsorption resin. Purification of the extract will be beneficial for qualitative and quantitative analyses of low levels of the compounds. The extract was purified by use of AB-8 macroporous adsorption resin with a column chromatographic mode, in which the AB-8 resin was used as an adsorbent and packed in a cylindrical glass column. Initially, an appropriate volume of 1.0 mg/mL solution (pH 2.0) of the extract was dropped into the column at a flow rate of 2 bed volume (BV)/h. Then, whole flavonoids were first adsorbed onto the AB-8 resin. The adsorbed flavonoids were eluted off the AB-8 resins by 70% ethanol at a 2 BV/h flow. Ethanol in the eluate was removed by a rotary thin film distillation method. The residue was then freeze-dried and the purified flavonoid extract powders from* J. regia* leaves were obtained.

### 2.5. HPLC-ESI-MS/MS Analysis

All analyses were performed using a Zorbax SB-C18 column (250 mm × 4.6 mm i.d., 5 *μ*m particle size) at 30°C with a binary phase at a flow rate of 0.3 mL/min. The mobile phase was a mixture of 0.1% (v/v) formic acid in methanol solutions (phase A) and 0.1% (v/v) formic acid in water (phase B). The gradient of phase A was 50%–72% (0–10 min) and then was held for 72% phase A to the end. The injection volumes of both the solution of purified flavonoid extract and the solution of the mixed standards were 5 *μ*L. The concentration of mixed standard substances was 8 *μ*g/mL. ESI was operated in negative ion mode. The working conditions for the ionization source were as follows: a capillary voltage of 4 kV, a source temperature of 350°C, and a nitrogen dry gas of 10 L/min and a high purity nitrogen (>99%) was used as nebulizing and collision gas.

Qualitative analysis of all compounds in the purified flavonoid extract was carried out by using HPLC-ESI-MS/MS. When the authentic standards were available, the compounds were identified by comparing their retention times and MS^2^ spectra with those of the reference standards. Otherwise, the compounds were proposed mainly based on the combined data of the following parameters including molecular ions, relative positions in the chromatogram, fragments released in MS^2^ experiments, and by comparison with the related literatures.

Quantitative analysis of the purified flavonoid extract was performed by HPLC-ESI-MS/MS in multiple reactions monitoring mode and monitoring the characteristic product ions selected from MS^2^ spectra. The compounds were quantified using an external standard method when commercial standards were available. The linear calibration curves were performed with at least five different concentrations of the related reference standards. Calibration curves were established by plotting the peak areas versus the corresponding concentrations of each injection standard substance. Other compounds were quantified using an internal standard method.

### 2.6. Validation of Assay

The limit of detection (LOD) and limit of quantification (LOQ) for each reference standard were determined at signal to noise (S/N ratio) of 3 and 10, respectively.

The precision of the method was evaluated by analyzing the mixed standard solution or real sample in six replicates. The precision was defined as the relative standard deviation (RSD, %) from six successive injections.

The accuracy of method was evaluated by recovery test. Appropriate amounts of the mixed standard solutions were added to the definite amounts of the solution of purified flavonoid extract. Then, the mixture was processed and analyzed using the proposed method. The recovery was calculated according to the following equation:

(1) Recovery(%) =(total  detection  amount−original  amountadded  amount)×100.

### 2.7. Cell Viability Assay

The cell viability assay using RAW264.7 cells was used to evaluate the cytotoxicity and sensitivity of the purified flavonoid extract from* J. regia* L. leaves according to the previous literature [7] with some modifications. RAW264.7 cells (1 × 10^5^/well) were seeded into a 96-well clear-walled flat bottomed plate (Nunc A/s, Denmark) in 100 *μ*L of growth medium. After being incubated at 37°C under 5% CO_2_ for 24 h, the growth medium was removed and the wells were washed with 100 *μ*L PBS twice. The cells were then treated with 100 *μ*L of the purified flavonoid extract solutions in different concentrations (0.1–1000 *μ*g/mL) for another 24 h. In order to avoid the effect of sample's color on the final reading, the medium (containing sample) was removed and wells were washed with PBS; then, 5 mg/mL MTT was added. After 4 h of treatment, an appropriate amount of DMSO was added to dissolve the MTT formation product. After the crystal was completely dissolved, the absorbance was measured at 570 nm using a microplate reader (SpectraMax M5, Molecular Devices, USA).

### 2.8. Intracellular Antioxidation Assay

The intracellular antioxidation was performed as previously described by Qian et al. [8] with some modifications. Briefly, RAW264.7 cells at a density of 1 × 10^5^/well were seeded into clear bottomed black 96-well plate (Nunc A/s, Denmark) in 100 *μ*L of growth medium and incubated at 37°C under 5% CO_2_. After 24 h, the medium was removed and the wells were washed with 100 *μ*L PBS twice. Triplicate wells were then treated for 1 h with 100 *μ*L of the purified flavonoid extract solutions with different concentrations (final concentration ranges, 25–500 *μ*g/mL) plus 10 *μ*M DCFH-DA. After this incubation, all wells were washed with PBS to remove any traces of DCFH-DA and 100 *μ*L of 300 *μ*M H_2_O_2_ was applied to the cells. The blank wells were treated with DCFH-DA and growth medium without H_2_O_2_, and the control cells were treated with DCFH-DA and H_2_O_2_. The outer wells were filled with PBS. The fluorescence in each well was measured at 5 min intervals over a 1 h period by a microplate reader with *λ*_ex_ 488 nm and *λ*_em_ 525 nm. Intracellular antioxidant activity was expressed as relative fluorescence units.

### 2.9. DPPH Radical Scavenging Activity

The DPPH free radical scavenging test was analyzed according to previous method described by Al-Duais et al. [9] with some modifications. An aliquot of 0.5 mL sample solution (2.5–20.0 mg/mL) was mixed with 4.0 mL of 0.2 mM DPPH ethanol solution. The mixture was left in the dark at room temperature for 30 min and absorbance was measured at 517 nm. In addition, 0.5 mL of ethanol with 4.0 mL of 0.2 mM DPPH ethanol solution was used as the control, and 0.5 mL of sample solution with 4.0 mL of ethanol was used as the blank. The radical-scavenging activity was calculated based on the following equation:

(2) $$\begin{array}{c} \text{Radical}\;\text{scavening}\;\text{activity}\;\left(\%\right)=100-\frac{A_{i}-A_{b}}{A_{c}}\times 100. \end{array}$$

*A*_*i*_ is absorbance of sample solution; *A*_*b*_ is absorbance of the blank; *A*_*c*_ is the absorbance of the control.

The results of the DPPH reduction in the purified flavonoid extract solution were linearized. The half maximum inhibitory concentration of DPPH radicals (IC_50_) was calculated according to the standard curve. All the results were measured three times and expressed in microgram per milliliter.

### 2.10. Statistical Analysis

Statistical analysis was undertaken using the general linear model procedure from Statistics Analysis System (SAS) (Version 9.2, SAS Institute Inc., Cary, NC, USA). All determinations were based on three replicate samples, and the results for content are shown as mean values. Differences between the means of sample were analyzed by the least significant differences test at a probability level of 0.05.

## 3. Results and Discussion

### 3.1. Qualitative and Quantitative Analysis

**Figure 1:**
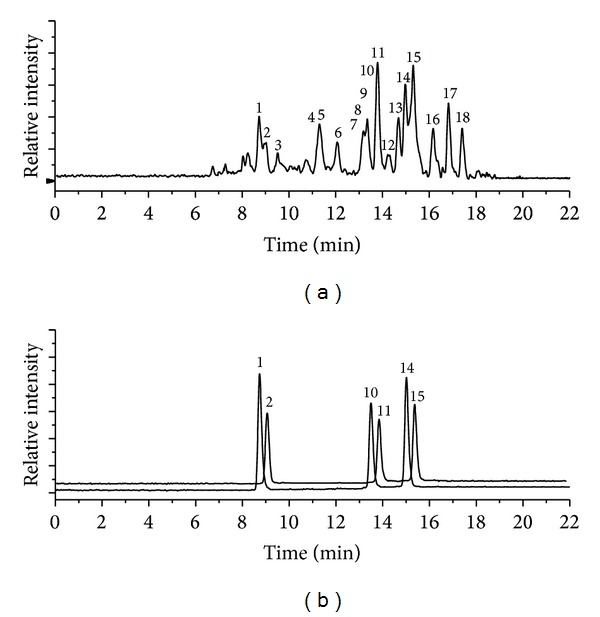
(a) Total ion chromatogram of the purified flavonoid extract from* Juglans regia *L. leaves and (b) total ion chromatogram of the mixture of reference substances. Peak numbers refer to Table 1.

The total ion chromatogram of the purified flavonoid extract from* J. regia* leaves is presented in Figure 1(a), and the one of the reference substances is shown in Figure 1(b). Based on the comparison fragmentation, MS and MS^2^ spectra with those of standards, and combining mass spectrometric cleavage of compounds and the related literatures, the results of qualitative and quantitative analysis of the seventeen compounds are shown in Table 1. In general terms, a coumarin compound, two phenolic acids, and fourteen flavonoids were determined including 5-O-caffeoylquinic acid, epicatechin, 3-p-coumaroylquinic acid, syringetin-O-hexoside, myricetin-3-O-glucoside, myricetin-3-O-pentoside, esculetin, taxifolin-pentoside, quercetin-3-O-glucuronide, quercetin-3-O-galactoside, quercetin-3-O-pentoside derivative, quercetin-3-O-xyloside, quercetin-3-O-arabinoside, quercetin-3-O-rhamnoside, kaempferol-O-pentoside, kaempferol-O-pentoside derivative, and kaempferol-O-rhamnoside. As far as we know, epicatechin, syringetin-O-hexoside, myricetin-3-O-glucoside, myricetin-3-O-pentoside, esculetin, taxifolin-pentoside, quercetin-3-O-glucuronide, kaempferol-O-pentoside, and kaempferol-O-rhamnoside are reported in this species for the first time. Some other phenolic acids were described to exist in Portuguese cultivars, namely, 3-O-caffeoylquinic acids, 4-p-coumaroylquinic acids, and p-coumaric acid, but these compounds were not detected in the present study.

Moreover, according to the results of quantitative analysis, the major components in* J. regia* leaves are quercetin-3-O-rhamnoside (453.11 *μ*g/g, dry weight), quercetin-3-O-arabinoside (73.91 *μ*g/g), quercetin-3-O-xyloside (70.04 *μ*g/g), kaempferol-O-pentoside derivative (49.04 *μ*g/g), and quercetin-3-O-galactoside (48.61 *μ*g/g), while in Portuguese* J. regia* leaves are quercetin-3-O-galactoside, 3-O-caffeoylquinic acids, quercetin-3-O-xyloside, 5-O-caffeoylquinic acids, and quercetin-3-O-arabinoside. The obvious differences existed in chemical composition of flavonoids and phenolic acids between Chinese cultivar and Portuguese cultivars.

### 3.2. Validation of Assay

The LOD and LOQ of the identified compounds were in the range of 1.85–9.93 ng/mL and 6.18–33.11 ng/mL, respectively. It could be seen that the LOQ values for all compounds were very low, which indicated the proposed method was sufficiently sensitive.

The precision of the method expressed in terms of RSD was lower than 5% from six replicate injections. The recoveries of these detected compounds were between 94.34% and 96.5% with RSD of 0.15–1.87%. The described above results indicated that the developed method for the quantitative analysis of flavonoids in* J. regia* leaves was suitable and accurate.

### 3.3. Cell Viability Assay

The reductases in living RAW264.7 cells could convert MTT into purple-colored formazan dye. Based on this reason, the number of living RAW264.7 cells could be expressed as enzyme activity level. Relative cell viabilities are 95.65% and the standard deviation of three replicates is ±4.59. It is difficult to find an obvious difference between high concentration group (1000 *μ*g/mL) and low concentration group (0.1 *μ*g/mL) of the purified flavonoid extract. This indicated that the purified flavonoid extract was almost no harm to RAW264.7 cells and the intake dose was also independent of the concentrations of the purified flavonoid extract in the ranges of 0.1 *μ*g/mL–1000 *μ*g/mL.

### 3.4. Intracellular Antioxidant Activity

**Figure 2:**
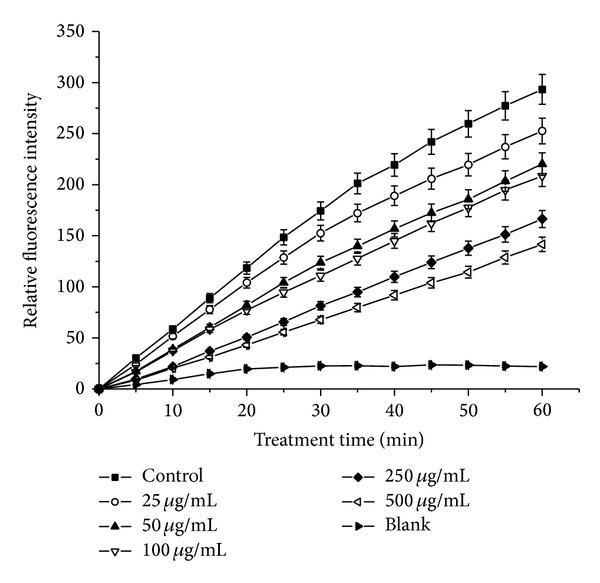
Intracellular radical scavenging activities of the purified flavonoid extract from leaves of* Juglans regia* L. in RAW264.7 cells. Data are reported as the mean ± SD of three replicates.

In this assay, DCFH-DA was used as a marker to evaluate the level of intracellular reactive oxygen species (ROS). Nonfluorescent DCFH-DA dye could freely penetrate into cells and was hydrolyzed into DCFH by intracellular esterase. Then, DCFH was oxidated to form fluorescent DCF by cellular ROS (generated by H_2_O_2_). So the fluorescence intensity of DCF could reflect the level of cellular ROS. As shown in Figure 2, the scavenging effects of the purified flavonoid extract on cellular ROS were compared with H_2_O_2_ nonstimulated blank and sample nontreated control groups. The treatment of the purified flavonoid extract could decrease the fluorescence intensity of DCF. This indicated that the purified flavonoid extract could be absorbed into cells and showed strong scavenging activity to ROS.

### 3.5. DPPH Radical Scavenging Activity

The DPPH free radical scavenging test provided an easy and rapid way to evaluate the antiradical activities of antioxidants. The antioxidants could scavenge the DPPH radicals, which caused a reduction of the absorption at 517 nm. A linearity relationship between DPPH radical scavenging activity and the concentrations of the purified flavonoid extract (*y* = 2.4453*x* − 4.8043; *R* ^2^ = 0.9994) was obtained. The values of radical scavenging rate of the purified flavonoid extract were in the ranges of 1.06%–56.33%. With the increase of the concentration of the extract, the scavenging activity was strengthened. The IC_50_ value was 22.41 *μ*g/mL.

## 4. Conclusions

The present study suggested that the methods herein employed were useful and accurate for characterization of the flavonoids in* J. regia* leaves. Seventeen compounds were identified, of which nine compounds including epicatechin, syringetin-O-hexoside, myricetin-3-O-glucoside, myricetin-3-O-pentoside, esculetin, taxifolin-pentoside, quercetin-3-O-glucuronide, kaempferol-O-pentoside, and kaempferol-O-rhamnoside are reported for the first time. Furthermore, the flavonoids in* J. regia* leaves were not cytotoxic, which did not significantly inhibit the growth of RAW264.7 cells. These flavonoids showed strong radical scavenging activities both intracellularly and extracellularly. The results provided a theoretical basis for a further study on the natural extracts from* J. regia* leaves.

## Figures and Tables

**Table 1:** The retention time, fragment ions, tentative identification, and quantification results of flavonoids and phenolic acids in *Juglans regia* L. Leaves.

Peak	*R*_*t*_ (min)	[M–H]^−^ (*m*/*z*)	MS^2^ (*m*/*z*)	Compound	Content (*μ*g/g)^e^	Reference
1	8.7	353	191, 179, 135	5-O-Caffeoylquinic acid (neochlorogenic acid)	29.72 ± 1.85	Std.
2	9.0	289	202.2, 123.0	Epicatechin^a^	8.86 ± 1.87	Std.
3	9.5	337	191, 163, 118.9	3-*p*-Coumaroylquinic acid	6.72 ± 0.13^b^	Ref. [10, 11]
4	11.1	507	327.3	Syringetin-O-hexoside^a^	5.99 ± 0.47^c^	Ref. [12]
5	11.2	339	158.6	Unknown^a^	38.63 ± 3.00^c^	
6	12.0	479	316	Myricetin-3-O-glucoside^a^	21.73 ± 1.69^c^	Ref. [12, 13]
7	13.1	449	316	Myricetin-3-O-pentoside^a^	8.88 ± 0.69^c^	Ref. [13, 14]
8	13.2	177	131.3, 115.2, 92.3	Esculetin^a^	9.79 ± 0.76^c^	Ref. [15, 16]
9	13.3	435	285, 150.8	Taxifolin-pentoside^a^	18.23 ± 1.42^c^	Ref. [17, 18]
10	13.5	477	300.9, 150.8	Quercetin-3-O-glucuronide^a^	4.30 ± 0.23^c^	Std.
11	13.8	463	300.0, 270.8	Quercetin-3-O-galactoside (hyperoside)	48.61 ± 0.11	Std.
12	14.3	433	300, 270.9	Quercetin-3-O-pentoside derivative	16.31 ± 0.24^d^	Ref. [1, 5, 11]
13	14.7	433	299.7, 271.1	Quercetin-3-O-xyloside	70.04 ± 2.77^d^	Ref. [1, 11, 19, 20]
14	14.9	433	301.0, 299.9, 270.8	Quercetin-3-O-arabinoside	73.91 ± 0.32	Std.
15	15.3	447	300.1, 284.2, 254.9	Quercetin-3-O-rhamnoside (huercitrin)	453.11 ± 11.65	Std.
16	16.2	417	285.0, 254.7, 226.9	Kaempferol-O-pentoside^a^	48.46 ± 3.78^c^	Ref. [21, 22]
17	16.8	417	284.7, 254.7	Kaempferol-O-pentoside derivative	49.04 ± 1.82^c^	Ref. [11, 21, 22]
18	17.4	431	285.2	Kaempferol-O-rhamnoside^a^	28.96 ± 1.26^c^	Ref. [23, 24]

^a^Compounds identified for the first time in *J. regia* leaves; ^b^expressed in equivalents of 5-O-caffeoylquinic acid; ^c^expressed in equivalents of quercetin-3-O-rhamnoside; ^d^expressed in equivalents of quercetin-3-O-arabinoside; ^e^mean ± RSD; Ref.: literature where the compound has been characterized by MS analysis; Std.: standard.
